# Risk Assessment of Heavy Metals in Selected Marine Fish Species of Gadani Shipbreaking Area and Pakistan

**DOI:** 10.3390/ani10101738

**Published:** 2020-09-24

**Authors:** Allauddin Kakar, Malik Tahir Hayat, Arshad Mahmood Abbasi, Arshid Pervez, Qaisar Mahmood, Umar Farooq, Tahir Ali Akbar, Shafaqat Ali, Muhammad Rizwan, Hamed A. El-Serehy, Mohamed M. Abdel-Daim

**Affiliations:** 1 Department of Environmental Sciences, COMSATS University Islamabad, Abbottabad 22060, Pakistan; kakar2k6@gmail.com (A.K.); mtahir@cuiatd.edu.pk (M.T.H.); amabbasi@cuiatd.edu.pk (A.M.A.); pervez@cuiatd.edu.pk (A.P.); 2 Department of Chemistry, COMSATS University Islamabad, Abbottabad Campus 22060, Pakistan; umarf@cuiatd.edu.pk; 3 Department of Civil Engineering, COMSATS University Islamabad, Abbottabad Campus 22060, Pakistan; tahiraliakbar@gmail.com; 4 Department of Environmental Sciences, Government College University, Faisalabad 38000, Pakistan; mrazi1532@yahoo.com; 5 Department of Biological Sciences and Technology, China Medical University, Taichung 40402, Taiwan; 6 Department of Zoology, College of Science, King Saud University, P.O. Box 2455, Riyadh 11451, Saudi Arabia; helserehy@ksu.edu.sa (H.A.E.-S.); abdeldaim.m@vet.suez.edu.eg (M.M.A.-D.); 7 Pharmacology Department, Faculty of Veterinary Medicine, Suez Canal University, Ismailia 41522, Egypt

**Keywords:** heavy metals, fish, seawater, risk assessment, Gadani shipbreaking

## Abstract

**Simple Summary:**

Protection of the coastal ecosystem from hazardous heavy metals is vital as it provides valuable habitat for numerous fish species and is a key resource for the coastal communities. Gadani shipbreaking is the third largest shipbreaking in the world, located on the coastline of Balochistan, Pakistan. The impact of this dismantling on the quality of the local fish species is still unknown. This is the first study to determine heavy metals’ content in fish and seawater of Gadani shipbreaking area. Metal accumulations in fish species both in gills and muscles ranged from 1.33 to 5.26 μg/g. Among trace metals, the level of Pb in all fish species was highest, followed by Ni, Mn, and Cd. However, all the analyzed fish species from the Gadani coast were found safe for human consumption, but there is a need for continuous monitoring of the coastal environment.

**Abstract:**

Gadani shipbreaking area, located on the coastline of Pakistan, is an important fish production area. In this study, levels of four metals (Ni, Pb, Cd, and Mn) in 148 muscle and gill samples of seven fish species (Small-scale terapon, Torpedo scade, Sicklefish, Saddle grunt, Gold silk seabream, Indian mackerel, Spotted sickle fish) and seawater samples, taken from 9 sampling sites in the shipbreaking area, were determined. In addition, multiple approaches were used to assess human health risks from fish consumption. Trace metal concentration in seawater ranged from 0.05 to 1.96 mg/L in shipbreaking vicinity and 0.03 to 0.97 mg/L in the reference site (Miani Hor). However, metal accumulations in fish species ranged from 1.33 to 5.26 μg/g. Among trace metals, the level of Pb in all fish species was highest, followed by Ni, Mn, and Cd. The bioaccumulation factors (BAFs) for both gills and muscles displayed the order: Mn > Cd > Ni > Pb. Estimated daily intake (EDI) values were below the tolerable daily intake (TDI). Based on target hazard quotient (THQ), the investigated fish species were safe regarding Pb and Mn (THQ < 1), while they may cause potential risk regarding Cd and Ni (THQ > 1). After comparison with maximum permissible limits, heavy metal concentration in the edible muscle tissues of all the analyzed fish species from the Gadani coast were found safe for human consumption.

## 1. Introduction

Seafood is a major source of diet for a large populous of the world, particularly coastal communities [1]. Fish is an important source of protein associated with many beneficial health effects [2]. Owing to its nutritional importance, its safety and quality is of prime importance [3]. Fish has extensively been studied around the world for heavy metals [4]. Ingestion of contaminated fish is an important route of human exposure to heavy metals [5]. Heavy metals are non-degradable; once they enter the ocean, they accumulate in organisms and bio-magnify in the apex predators, and then transfer the toxic pollutant load through the food web [4]. This in turn lowers the quality of seafood and is a potential human health risk. Fish consumed worldwide are found to be highly contaminated by heavy metals [6].

The shipbreaking industry has imported millions of tons of toxic waste to the coastal beaches of South Asia [7]. These end-of-life ships’ waste is of different nature, like oil, asbestos, organotins, Polycyclic Aromatic Hydrocarbons (PAHs), Polychlorinated Biphenyls (PCBs), and heavy metals. These often get mixed with beach sediment and seawater, which in turn has a negative impact on the coastal environment and biodiversity [8].

Shipbreaking activities have impacted the marine environment and various studies have already expressed concerns [9]. The metals released into the water system are absorbed and deposited by suspended sediments [10]. This reduces the concentration of heavy metals in the water column and makes surface sediments a reservoir for particle-related pollution [11]. Due to the bioaccumulation and bioconcentration process, the influence of heavy metals can be detected on land via the food web [11]. It is important to constantly monitor fish species for concentration of heavy metals as it provides a good indication of pollution status [12]. Contaminated fish is an important source of heavy metal exposure in humans. Therefore, the assessment of metal content in the organs and tissues of fish is of high significance.

Pakistan has a coastline of 990 km, comprised of two parts: the coast of Balochistan (745 km) and the coast of Sindh (245 km) [13]. Balochistan coastline is mainly unpopulated, and its beaches are one of the cleanest in the world. This coastal region provides valuable habitat for numerous species and is a key resource for the coastal populous [14]. The coastal people rely primarily on fisheries and boat making for their livelihood [15].

Currently, Pakistan is one of the world’s largest shipbreaking countries and ranks third in the world in terms of scrapped tonnage and in number of ships, followed by Bangladesh and India [15]. This sector has been ignored so far, by both the provincial and federal government [16].

End-of-life tankers, bunkers, and container ships are beached on designated slots for almost two to three months to be dismantled. Hundreds of accidents have led to severe environmental as well as human health issues in the region. According to the World Bank usual scenario-based study, the projected accumulation of heavy metals that will remain at the yards or in beach sediments for 2010–2030 will be 22 tons [16]. The area contains 314 plots of different sizes—135 of them are active shipbreaking yards. The beach of Gadani is sandy and the water level is deep with a tidal range between 1 and 3 m.

The Balochistan coastal zone, up to about 20 km inland from the coast, is delineated by the Makran Coastal Mountain Range (MCMR). MCMR runs parallel to the coast and separates it physically, socially, and economically from the rest of the province. Balochistan’s coastal water is defined by a narrow continental shelf, mostly 15–50 kilometers wide at the 200 meter isobaths. From here, the continental slope dips sharply, delimiting an extensive, deep offshore zone.

The sole large island along the Balochistan coast is Astola (Haft Talar), 7 square km, 39 km from Pasni. Astola is uninhabited. It is endowed with 29 different species of corals, endangered green and hawksbill turtles, and is a breeding ground for many water-birds. It is one of the four Ramsar Convention sites in Balochistan. Corals are also found at 9 locations along the Balochistan coastline, e.g., they have recently been discovered at Daraan and Gunz, which add to the ecological importance of the coastal areas of the province.

As a result of monsoon dynamics and strong seasonal upwelling of nutrient-rich water from the depths along the narrow continental shelf, there is high surface productivity in the Arabian Sea and the area is known to be rich in marine biodiversity. The Balochistan coastal zone is rich in marine fisheries, which include about 350 different species. Some 240 are demersal fish, 50 are small pelagic, 10 are medium-sized pelagic, and 18 are large pelagic fish. In addition, there are 15 species of shrimps, 12 of squid/cuttlefish/octopus, and 5 species of lobsters.

The shipbreaking industry has introduced hazardous waste to the coastline of Pakistan. No comprehensive study has yet been carried out to determine the contamination level and its impact on water and marine fish and associated human health from the consumption of fish in the Gadani coastal area. Therefore, the aim of this study is to investigate the accumulation of heavy metals in water and in fish along the shipbreaking area, and human health risk from the toxicity of selected heavy metals owing to the ingestion of fish.

## 2. Materials and Methods

### 2.1. Study Area

**Figure 1:**
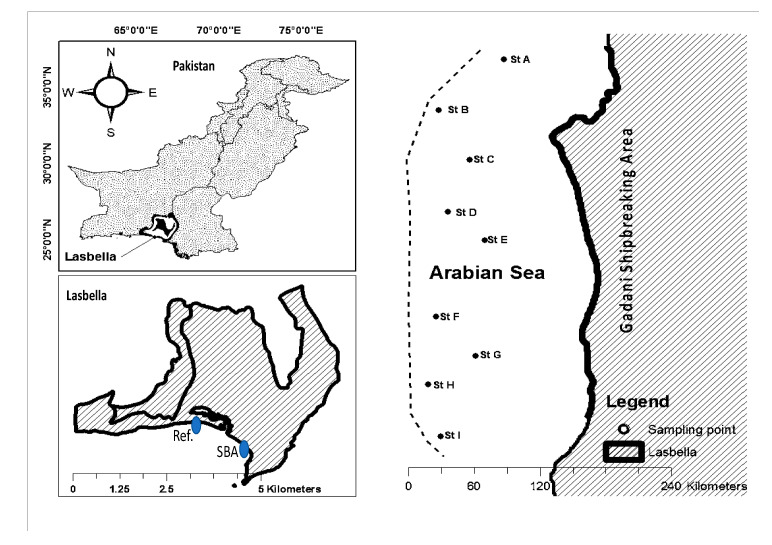
Map showing the location of sampling stations (St) at Gadani shipbreaking area (St A–I).

Gadani is a small town situated in Tehsil Hub of Baluchistan’s Lasbela district, about 50 km to the north-west of Karachi [14]. The coordinates of the sampling area are Latitude: 25° 07′ 6.71″ N and Longitude: 66° 43′ 47.46″ E (Figure 1). It is known for its golden sandy beach. From the Gadani town, around 10 km down to the east is a stretch of land (about 10 km long), where Pakistan’s shipbreaking industry is located [16]. The climatic condition in the region is arid, with annual rainfall ranging from 100 to 200 mm with a mean annual temperature exceeding 25 °C. The area is dominated by dry periods throughout the year.

### 2.2. Collection of Samples

Seawater was collected from two sampling sites, one from the unpopulated reference site around 50 km away from the shipbreaking site (Miani Hor), and the other from the shipbreaking adjacent area (Figure 1).

For water sampling, acid-washed polyethylene bottles were used. The bottles were immersed from the boat about 10 cm below the surface seawater and filled. Samples were taken from a 10 km horizontal area along the shore and 1 km vertically from the Gadani shipbreaking area. Samples were kept with ice and transported to the laboratory. Samples were acidified (pH < 2) with concentrated HNO_3_ (*v/v*) and passed through membrane filters (0.45 μm) before analysis.

Seven locally consumed fish species (Small-scale terapon, Torpedo scade, Sicklefish, Saddle grunt, Gold silk seabream, Indian mackerel, Spotted sickle fish) were collected in the vicinity of the shipbreaking area randomly by hiring a special boat. The fish species were documented according to their length, feeding habits, habitat, weight, and their importance in the local fishery (Table 1). Collected fish species were immediately preserved in an icebox and transferred to the laboratory.

The study design was duly approved by the Biosafety and Bioethical Committee of the COMSATS University Islamabad, Abbottabad Campus, which allowed the use of fish species for the current research design according to international standards on the use of animals for research purposes (AHBP-HEC 2015-20). Before the experiments, the proposal was submitted to the Bioethical Committee for review and consideration. The Bioethical Committee assessed the research plan of the current research and approved it based on the following international standards:

1. As fish are edible, and the killing of fish was indispensable, hence killing of fish samples was allowed to determine the heavy metals’ concentration in various body parts.
2. The killing of fish samples followed procedures to avoid distress and caused rapid loss of consciousness without pain until death.
3. After the experiment, the remains of body parts were properly disposed of in a landfill.

### 2.3. Physiochemical Analysis and Samples’ Treatment

Water temperature, pH, salinity, and Electrical conductivity (EC) were measured through advanced portable meters. The stored fish species were dissected for muscles and gills with stainless-steel equipment [17]. Selected fish were dissected for about 1.0 g of gills and muscles. The samples were washed with deionized water, weighed, and stored in zip bags at −18 °C until chemical treatment.

The muscular tissues were taken from the tail, dorsal, and abdomen portion of the body and a composite sample was made. Muscles are the main route of human exposure to heavy metals. Whereas, gill fragments are important to monitor water quality due to their water exchange characteristics. Brachial arches were collected from both sides [3,18].

For acid digestion, 4 mL of nitric acid (65%) and 1 mL of per-chloric acid (35%) were used. Samples were transferred to digestion tubes and pre-digested overnight in digestion solution. The tubes were then inserted into a heater for approximately 2 h at 275 °C. Once cooled, the samples were diluted to 25 mL with double-distilled water. Blanked and spiked samples were treated in the same way. Samples were then filtered (0.45 μm) and kept in acid-treated plastic bottles until analysis [19,20].

### 2.4. Analytical Procedures and Quality Control

Samples of water and biota were quantified by Graphite Furnace Atomic Absorption Spectrometry (GFAAS) (PerkinElmer-AAnalystTM700) for the selected trace metals, including cadmium (Cd), manganese (Mn), nickel (Ni), and lead (Pb). All the standard solutions and reagents were of ACS (American Chemical Society) grade with a high degree of purity (≥ 95%) [21,22].

For accuracy, the calibration line method was employed by maintaining optimum analytical conditions. Standards were made for each metal from the 1000 ppm stock solution of Perkin Elmer stock standards. From dilutions, 0.5 mg/L was used for a recovery check. For the calibration curve, the concentrations chosen were low, medium, and high (depending upon the calibration points). No standard reference material was available for this study. Blank samples were spiked with analyte to check the calibration of the instrument as this also gives a good indication of accuracy [21]. The concentrations added and the concentrations found were noted [22]. The samples were then tabulated and quantified to determine the percent recovery of the analyte [23]. The recoveries of different analytes were Pb: 120%, Cr: 96%, Ni: 116%, Cd: 80%, and Mn: 90%.

There are various methods for determining LOD (Limit of Detection) and LOQ (Limit of Quantification) [24]. The blank determination method was used to assess LOD and LOQ [23]. This method is based on taking the standard deviation of 20 or more blank readings using the following equations:

LOD = Xb1 + 3Sb1(1)

LOQ = Xb1 + 10Sb1(2)

where Xb1 = average blank concentration, and Sb1 = standard deviation of the blank concentrations. Although this is a quick and simple method, there is no evidence to prove that the low analyte concentration is producing a signal that can be reliably distinguish from zero concentration (Blank sample).

### 2.5. Human Exposure Assessment

#### 2.5.1. Bioaccumulation Factor (BAF)

BAF is the relative proportion of metal concentration in an organism to that of metal concentration in water [25]. BAF shows a correlation of metal uptake in water in relation to other sources. Exposure is assumed to be through all routes (i.e., dietary, dermal, transport through respiratory surface). Unlike the bioconcentration factor, the bioaccumulation factor is usually estimated under field conditions. Bioaccumulation combines biomagnification and bioconcentration. BAF was calculated according to the following equation [26]:

BAF = Metal content in organism (CB)/metal content in water (Cw).(3)

Heavy metal sorption with dissolved and particulate matter may reduce metal bioavailability in water column. However, BAF can also be expressed for freely dissolved chemicals in water. Hence, it has a universal applicability. The unit for BAF is L/kg and metal content in fish and water is expressed as mg/kg (ww) and mg/L, respectively.

#### 2.5.2. Consumption Data

The current study has taken an average fish consumption of a selected population (Balochistan). The fish consumption rate was documented from the National Bureau of Statistics (Pakistan) and FAO (Food and Agriculture Organization) international consumption surveys (5.81 Kg/capita/annum) [27].

#### 2.5.3. Estimated Daily Intake (EDI)

For EDI, fish consumption was multiplied with average concentration of heavy metals in the muscles of fish. Then, this was divided by an average body weight. Only the data of muscles were used in calculating all indices. The equation for estimated daily intake reported by [28] is as expressed in Equation (4):

EDI = FIR × C/Bw(4)

In Equation (1), the EDI stands for average daily dose over a lifetime through the consumption of fish, while FIR is the food ingestion rate in kilograms per day (14.1 × 10^−3^ kg/day), and C is the average heavy metal concentration in fish muscles (μg/g). Lastly, Bw shows the average body weight, considered as 52 kg.

#### 2.5.4. Target Hazard Quotient (THQ)

Non-cancer risk assessment is estimated by THQ. In THQ, the dose of the consumer is divided by a reference dose. A THQ value less than 1 is of less concern. RfDs (Reference Dose) are provided by international and national agencies [29].

The equation for THQ according to [30] is as follows (Equation (5)):

THQ = CM × Cf × IR × ED × EF/Bw × ATn × RfD × 10^−3^ (5)

#### 2.5.5. Hazard Index (HI)

The HI is the sum of all hazard quotients for each heavy metal [29]. The equation is as follows (Equation (6)):

HI = THQ (Pb) + THQ (Cd) + THQ (Ni) + THQ (Mn)(6)

#### 2.5.6. Target Cancer Risk (TR)

TR is estimated for those metals that have a carcinogenic slop factor, and, on exposure, have a probability to develop cancer. For Ni, carcinogenic slop factor values are available (Ni = 0.00009 ASTDR) (Agency for Toxic substances and Disease Registry) [29,31]. The acceptable level ranges from 10^−4^ to 10^−6^ for lifetime cancer risk. The equation to estimate TR is as follows (Equation (7)):

TR = ED × EF × CF × IR × RfC × C/Bw × ATc × 10^−3^ (7)

### 2.6. Statistical Analysis

Pearson correlation matrix (r) of the metal accumulation in muscles and gills with seawater was employed at the 0.05 significance level.

## 3. Results

### 3.1. Physico-Chemical Analysis and Heavy Metal Concentration in Seawater

The ranges of pH, conductivity (μS/cm), salinity (ppt), and temperature (°C) in the shipbreaking area were from 7.00–8.00, 36,500–38,500, 36.3–37.4, and 20.1–22.90, respectively (Table 2). Whereas, in the reference site (Miani Hor), the pH, conductivity (μS/cm), salinity (ppt), and temperature (°C) values ranged from 7.51–7.96, 35,800–36,500, and 38.00–39.00, respectively. There was a clear difference between the values of these two sites. Compared to the reference site, conductivity was low in the shipbreaking area owing to the continuous discharge of oil.

The selected heavy metal concentration in the shipbreaking area and Miani Hor is listed in Table 2. In the shipbreaking area, the concentration of Pb, Cr, Ni, Cd, and Mn in seawater ranged from 1.81–2.11, 0.017–0.34, 0.49–1.235, 0.18–0.38, and 0.027–0.25 mg/L, respectively. In Miani Hor, the concentration of Pb, Cr, Ni, Cd, and Mn ranged from 0.92–1.01, 0.02–0.11, 0.71–0.95, 0.20–0.26, and 0.01–0.06 mg/L, respectively. The average concentration of selected heavy metals in the shipbreaking area (0.6934 mg/L) was more than that in Miani Hor (0.4272 mg/L).

### 3.2. Heavy Metals’ Content in Fish

The heavy metals’ content in fish species are compiled in Table 3. The average Pb concentrations in all fish species (gills and muscles) was 4.51 μg/g. The highest concentration was found in the gills of Spotted sickle fish (9.44 μg/g) and the lowest concentration was in the muscles of Gold silk seabream (0.155 μg/g). The mean Pb concentration (gills and muscles) in all fish species displayed the order (μg/g): Spotted sickle fish (7.66) > Small-scale terapon (7.39) > Sickle fish (7.32) > Saddle grunt (4.9) > Torepedo scade (4.0) > Gold silk seabream (2.61) > Indian mackerel (2.02) (Table 3).

The average Ni concentration in gills and muscles, in all examined fish species, was 4.99 μg/g. Ni minimum concentration was found in the muscles of Saddle grunt (0.125 μg/g) and maximum concentration was found in the gills of Torepedo scade (13.85 μg/g). The mean of Ni concentration (gills and muscles) in all analyzed fish species followed the order: Small-scale terapon > Spotted sickle fish > Gold silk seabream > Sickle fish > Saddle grunt > Torpedo scade > Indian mackerel. The level of Ni in muscles in all analyzed fish species exceeded the permissible limit for human consumption.

The mean Cd concentration was lower than all analyzed metals in selected fish species. The average concentration for gills and muscles in all individual fish was 1.37 μg/g. The highest level was observed in Torpedo scade (4.18 μg/g) and the lowest concentration was also recorded in Torpedo scade (0.075 μg/g). There was no significant difference among fish species except Gold silk seabream. The average Cd load (gills and muscles) in fish species was decreased by the order: Sickle fish > Spotted sickle fish > Torpedo scade > Gold silk seabream > Small-scale terapon > Saddle grunt > Indian mackerel.

The Mn concentrations in all fish species ranged from 0.73 to 7.69 μg/g. The highest level was observed in Gold silk seabream and the lowest was recorded in Sickle fish. The average Mn concentration (gills and muscles) in all selected fish species was 2.86 μg/g. The mean Mn concentration in each individual fish were in the order: Gold silk seabream > Indian mackerel > Saddle grunt > Small-scale terapon > Torpedo scade > Spotted sickle fish > Sickle fish. Mn concentrations in muscles of selected fish species were higher than the recommended permissible limits.

### 3.3. Bioaccumulation Factor

**Figure 2:**
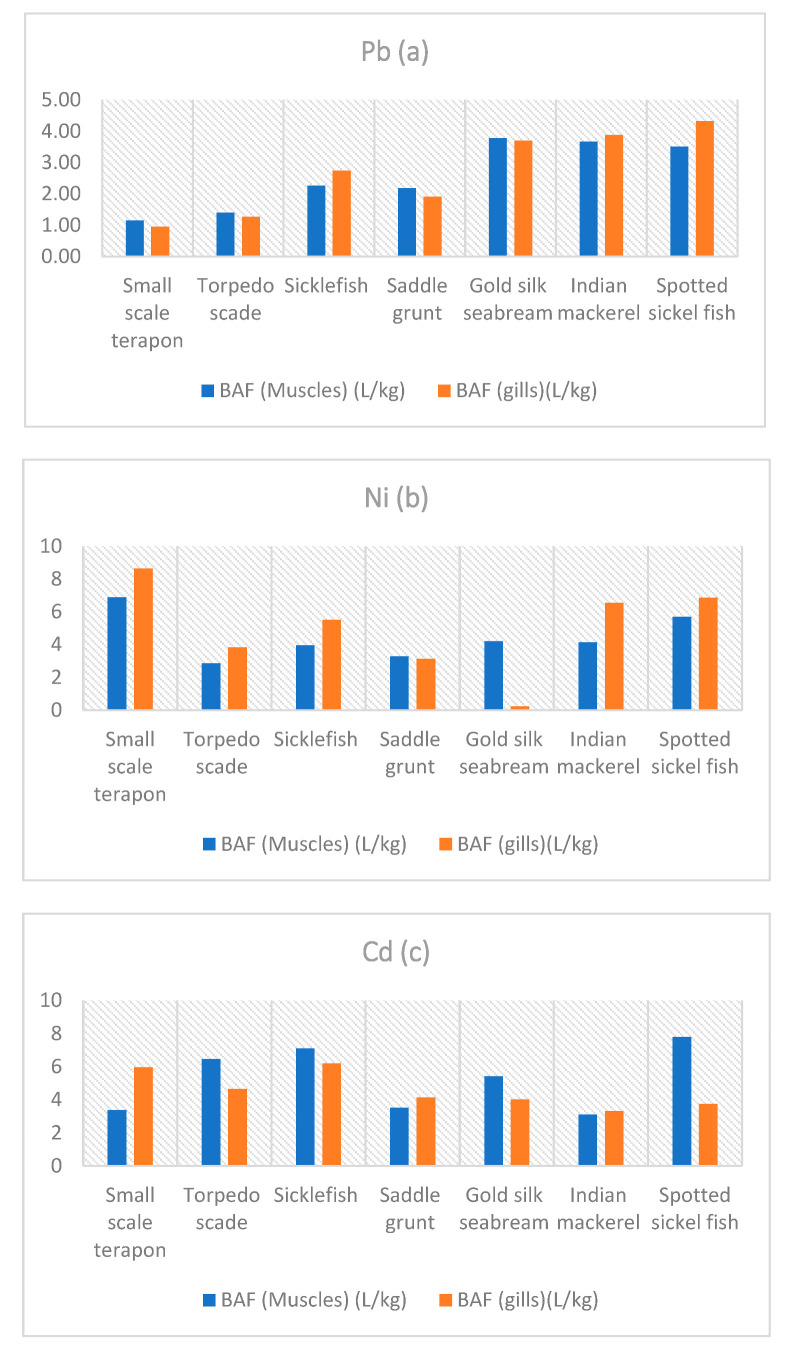

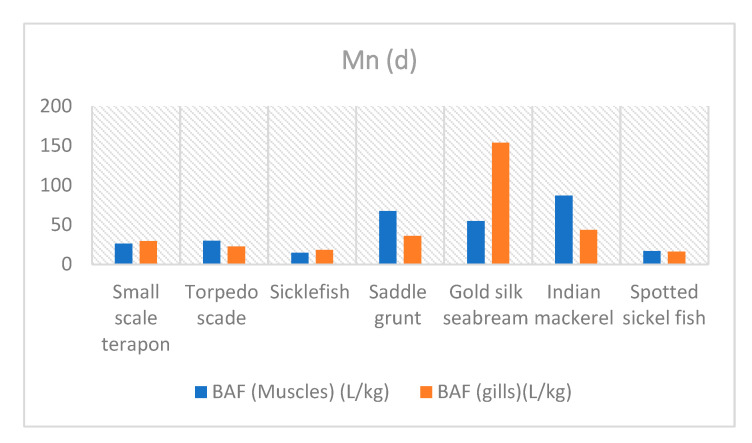
Bioaccumulation factors (**a**–**d**) in all the selected fish species.

The bioaccumulation factors of selected heavy metals in fish were calculated and summarized in Figure 2. Mn showed the highest BAFs in the gills of Spotted sickle fish and Gold silk seabream. The BAF values for both gills and muscles displayed the order: Mn > Cd > Ni > Pb. The BAFs values of this study are in line with other studies (Figure 2).

### 3.4. Risk Assessment

#### 3.4.1. Estimated Daily Intake (EDI)

Estimated daily intakes for each selected metal in individual fish are presented in Table 4. The mean EDI intakes for Pb, Ni, Cd, and Mn were 0.0014, 0.0013, 0.0004, and 0.0006, respectively. The mean EDI values were in order of Pb > Ni > Mn > Cd.

#### 3.4.2. Target Hazard Quotient (THQ) and Hazard Index (HI)

THQ values for selected heavy metals in individual fish species are presented in Table 5. The current study showed that THQ values for Pb and Mn in all fish species were less than unity. Whereas, Cd showed the highest THQ values for all fish species, and Ni for Small-scale terapon (1.07). Based on THQ, and single metal ingestion, fish species were safe regarding Pb and Mn (THQ < 1) and may cause potential risk regarding Cd and Ni (THQ > 1).

#### 3.4.3. Target Cancer Risk (TR)

TR values for Ni in Small-scale terapon, Torpedo scade, Sickle fish, Saddle grunt, Gold silk seabream, Indian mackerel, and Spotted sickle fish, ranged between 1^−5^ to 6^−6^ (Table 5), which is within the acceptable range.

## 4. Discussion

The physiochemical properties determine the overall health of any aquatic system [37]. Shipbreaking severely affects the physiochemical properties of seawater due to the continuous discharge of liquid waste, such as salt, caustic agent, paints, and various other contaminants [7]. Several studies reported high pH, turbidity, TSS (Total Suspended Solids), TDS (Total Dissolved Solids), ammonia, and high EC in adjacent seawater [7,38,39]. Metals released into the aquatic system are adsorbed by suspended sediments and settle down. This results in the lower concentration of heavy metals in the water column [40]. The accumulated heavy metals in sediments are released into the water column by certain variables, like redox potential, altering pH, mobilization of benthic biota, and sediment re-suspension [41]. The results of this study were in accordance with other regional studies conducted on shipbreaking areas reported in Table 6. Fish uptake heavy metals by two pathways: through the digestive tract by diet exposure and through the gills’ surface by water exposure [42]. The correlation study showed that there is a positive correlation between metal concentration in seawater and muscles (*p* < 0.05) and a negative correlation between seawater and gills (*p* > 0.05). It is important to constantly monitor seawater for concentration of heavy metals as it provides a good indication of pollution status [43].

The average Pb concentration for all fish species was higher than the study conducted in the Bay of Bengal, Palk bay, India, Turkish Sea, Mian kaley, Lake Iran, and lower than the study conducted in the Mediterranean Sea (7.33–9.11 μg/g), Jedda Coast, and Saint Martin Island. Whereas, the Pb concentration in examined fish species agreed with the reported studies for the Gulf of Aqaba and Bangshi river (Table 6). The Pb concentration was also higher than the permissible limits (0.5 μg/g—FAO, 2 μg/g—USEPA (United States Environmental Protection Agency), 2 μg/g—WHO (World Health Organization) [34,35,36]. This high level of Pb may be attributed to the highly contaminated sediments of the shipbreaking area. In Chittagang, it was reported that the Pb concentration in sediments ranged from 4232 to 5733 mg/kg [44]. Pb is mainly released from paints, batteries, and electrical equipment, during the ship dismantling process into the beach sediments that are carried away by the waves and tides. This then makes its way into the food chain. Thus, this high level of Pb in selected fish species may be attributed to a high level of Pb in seawater (Table 2). Thus, the continuous mobilization of contaminated sediments may be a source for metal uptake by the adjacent biota. Through ingestion of contaminated fish, Pb toxicity can inhibit or mimic the actions of calcium, and in the same way, Pb also has an affinity for sulfhydryl group that in turn disturbs multiple enzyme systems.

Ni levels in the muscles were higher than those from the Turkish seas [56]. And the South East Coast of India [57], and lower than the studies reported in the South West coast of India and the Red Sea [45,49].

The Cd level was lower than those observed in the fish species of the Santa Maria Bay (1.52–14.09) [58], while higher than the values displayed for other fish in the Red Sea [59], the SE coast of India [57], and the Bay of Bengal [60]. However, the results of Cd in analyzed samples were in line with the study conducted in the oil-spilled area and the Red Sea [61]. The mean Cd concentrations in the muscles of individual fish species were in the range of permissible limits [62].

The Mn concentration in this study was lower than the values observed for Mn in the fish species reported from the Red Sea and the Bay of Bengal. However, the Mn concentration exceeded the values observed for other fish species in the Gulf of Aqaba [47] and the SE coast of India [57].

**Figure 3:**
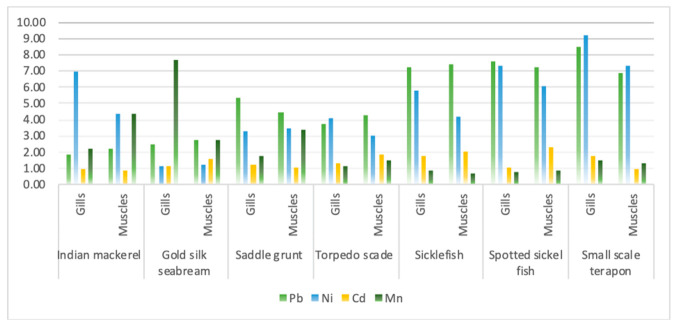
Comparison of heavy metals’ accumulation in fish species.

Variation between fish species regarding metal accumulation was shown in Figure 3. There was a slight difference between metal accumulation in gills and muscles of different fish species. Among the observed fish species, Pb was highly accumulated, followed by Ni, Mn, and Cd. As mentioned earlier, fish uptake heavy metals by two pathways: through the digestive tract by diet exposure and through the gills’ surface by water exposure. Gills and muscles are important to monitor as they reflect the surrounding environment. This study correlated metal concentration in aqueous medium with that of metal concentration in gills and muscles. The correlation study showed that there is a positive correlation between metal concentration in seawater with muscles and a negative correlation of metal concentration in seawater with gills. Muscles are important for dietary exposure.

The metals released into the water system are absorbed by suspended sediment, and then settle down [10]. This causes a lower concentration of heavy metals in the water column and makes surface sediments a reserve for pollution [63]. Bottom fauna depends on bottom sediments in terms of habitat and food source [4]. Thus, the metals associated with the particles are a source of pollution [11]. Fish that have been affected are an important way of exposing heavy metals to humans. Therefore, this poses a risk to human health.

Bioaccumulation refers to an organism’s intake ability of chemicals from the aqueous medium. Bioconcentration is different from bioaccumulation in a way that bioconcentration depends only on water exposure, while bioaccumulation considers both dietary and water exposure [64]. Bioconcentration is mainly done for lab data and bioaccumulation for field data, as one cannot be sure that the heavy metals’ concentration in fish is only because of water in the field. The value of BAF > 1 generally declares that the biota can potentially accumulate heavy metals in their body, but this becomes significant when the value exceeds 100 or more. BAF values greater than 1000 indicate a hazard, as declared by many regulatory agencies. This value has its origin from non-polar compounds. The BAF value > 1000 signifies slow and significant accumulation, which means that there is a potential for chronic effects and a chance for tropic transfer. BAF of Mn was greater than 100 and less than 300, while the average Ni, Pb, and Cd BAF values were less than 100, showing potential accumulation.

The mean of all EDI was below the tolerable daily intake (TDI) (0.0015) [30,34,35]. The mean EDI values compared with the TDI values showed that there is no detrimental health risk from the consumption of selected fish in the study area.

THQ is actually a model for relating the average chronic daily intake of contaminants in diet with the reference dose, calculated by International and National Agencies (ASTDR, IRIS, EPA (Environment Protection Authority)). THQ > 1 signifies a potential risk, whereas THQ < 1 means there is no risk, or a minimal potential risk that is negligible. THQ is actually suggested for non-carcinogenic metals. The human population is exposed to the combined impact of contaminants. An accumulative risk index including selected heavy metals is required for all individual fish species. This is called the hazard index (HI) [30], and it is the sum of all THQs for each individual fish. The HI values for all individual fish species were greater than unity and are presented in Table 5. Based on HI values, Small-scale terapon, Torpedo scade, Sickle fish, Saddle grunt, Gold silk seabream, Indian mackerel, and Spotted sickle fish may cause potential health risks. The continuous exposure from these metals through the consumption of selected fish species may cause chronic health effects.

This study considered only Ni, owing to its availability of carcinogenic slop factor, for the TR Index [30]. The TR value is calculated for those metals that have a known carcinogenic effect upon lifetime exposure. TR values for Ni are listed in Table 5.

TR values > 1 in a million (10^−6^) are considered a risk, and this is unacceptable by the USEPA standards (2000). However, the acceptable level may vary somewhere else in accordance with national standards and environmental policies and may be as high as 10^−4^. Risks that lie between 10^−4^ to 10^−6^ are considered acceptable [29]. The TR values indicate that there is a low or minimal carcinogenic risk from the consumption of the examined fish species.

## 5. Conclusions

In this study, concentrations of selected metals in seven fish species collected from different sampling sites in the vicinity of the Gadani shipbreaking area were determined. Fish species showed diminutive variability in their ability to accumulate heavy metals in gills and muscles. Among analyzed trace metals, the maximum accumulation occurred for Pb in fish species, followed by Ni, Mn, and Cd. From the human health point of view, the average THQ values for Ni and Cd exceeded 1, which suggests that there may be a potential non-carcinogenic health risk for humans from these two metals. Results also showed that estimated daily intake (EDI) values were less than the tolerable daily intake values (TDI). The target cancer risk (TR) values for Ni in all fish species were below the 10^−6^ threshold. Hence, there is no cancer risk. The maximum allowable daily consumption rates were high enough to safeguard human health. In conclusion, this study indicated that the consumption of seven fish species from the Gadani shipbreaking area is safe for human health. It is recommended that seawater and biota should continuously be monitored for assessment.

## Figures and Tables

**Table 1:** Morphometric characteristics of analyzed fish species.

Scientific Name	Common Name	Habitant	No. Of Samples	Average Weight (g)	Length (cm) (Total Lenght)
***Terapon puta Cuvier***	Small-scale terapon	Pelagic	12	45.359 ± 5.4	16
***Megalaspis cordyla)* (IUCN red list)**	Torpedo scade	Pelagic	8	861.826 ± 4.3	25
***Drepane***	Sicklefish	Benthic	12	635.029 ± 6.2	25
***Pomadasys maculatus* (IUCN red list)**	Saddle grunt	Pelagic	10	90.718 ± 5.3	16
***Acanthopagrus australis***	Gold silk seabream	Demersal/coastal waters	8	1859.73 ± 4.6	23
***Rastrelliger kanagurta***	Indian mackerel	Shallow coastal waters	12	226.796 ± 6.6	30
***Drepane punctata***	Spotted sickle fish	shallow coastal waters	12	635.029 ± 6.3	25

IUCN: International Union for Conservation of Nature.

**Table 2:** Heavy metals and physicochemical parameters of seawater in Gadani shipbreaking area.

Parameters	Shipbreaking Zone (Gadani)		Reference Zone (Miani Hor)		Other Regional Shipbreaking Areas		
	Min–Max	Average ± SD	Min–Max	Average ± SD ^1^	Average ^a^	Range ^b^	Average ^c^
Pb (mg/L)	1.81–2.11	1.96 ± 0.07	0.92–1.01	0.97 ± 0.03	0.07	0.9–1.05	1.77
Cr (mg/L)	0.017–0.34	0.107 ± 0.08	0.02–0.11	0.066 ± 0.04	0.04	0.36–0.45	0.678
Ni (mg/L)	0.49–1.235	1.06 ± 0.15	0.71–0.95	0.84 ± 0.09	0.08	0.41–0.72	0.696
Cd (mg/L)	0.18–0.38	1.06 ± 0.15	0.20–0.26	0.23 ± 0.02	0.0034	0.04–0.06	0.446
Mn (mg/L)	0.027–0.25	0.05 ± 0.04	0.01–0.06	0.03 ± 0.02	0.48	ND ^2^	4.36
Temperature (°C)	20.1–22.90	21.80 ± 1.31	19.98–22.89	22.13 ± 1.44			
Conductivity (μS/cm)	36,500–38,500	37,280 ± 0.45	35,800–36,500	36,210 ± 0.27			
pH	7.00–8.00	7.76 ± 0.06	7.01–8.00	7.89 ± 0.07			
Salinity (ppt)	36.3–39.7	37.4	38–39	38.5 ± 0.3			

^1.^ Standard Deviation. ^2.^ Not Detected (a) [31], (b) [32], (c) [33].

**Table 3:** Mean ± standard deviation (SD) of selected heavy metals (μg/g) (Wet Weight) in fish caught in the vicinity of Gadani shipbreaking area.

	Pb (μg/g)		Ni (μg/g)		Cd (μg/g)		Mn (μg/g)	
	Gills	Muscles	Gills	Muscles	Gills	Muscles	Gills	Muscles
Indian mackerel								
Mean	1.87	2.24	6.95	4.38	0.96	0.9	2.18	4.34
SD	0.77	1.18	5.02	1.62	0.64	0.27	4.40	4.52
Minimum	0.89	1.15	1.41	1.54	0.37	0.37	0.01	1.3
Maximum	2.85	4.04	13.7	6.2	2.16	1.12	11.16	10.59
Gold silk Seabream								
Mean	2.49	2.74	0.241	4.47	1.165	1.57	7.69	2.73
SD	1.29	1.07	3.07	2.60	0.38	1.46	12.86	8.90
Minimum	0.61	0.15	1.17	1.24	0.57	0.84	0.46	0.22
Maximum	4.65	4.99	11.86	12.37	2.02	1.7	44.6	44.4
Saddle grunt								
Mean	5.37	4.43	3.32	3.47	1.2	1.019	1.8	3.37
SD	1.42	1.31	2.05	2.63	0.80	0.45	1.16	4.75
Minimum	3.1	3.09	0.37	0.12	0.19	0.26	0.82	0.55
Maximum	7.34	7.13	5.84	8.14	3.34	1.56	3.94	17.64
Torpedo scade								
Mean	3.72	4.27	4.06	3.03	1.35	1.87	1.12	1.49
SD	2.27	1.86	3.53	2.43	0.44	1.18	0.62	1.07
Minimum	0.26	0.375	1.65	0.16	0.84	0.07	0.64	0.66
Maximum	7.05	6.89	13.85	7.32	2.24	4.19	2.84	3.64
Sicklefish								
Mean	7.59	7.40	5.83	4.18	1.8	2.06	0.91	0.73
SD	1.16	1.55	2.34	2.21	0.63	0.50	0.63	0.34
Minimum	5.87	4.15	9.4	6.81	0.84	1.17	0.09	0.28
Maximum	8.96	8.78	1.84	0.2	2.82	2.73	1.66	1.38
Spotted sickle fish								
Mean	8.45	6.87	7.28	6.041	1.09	2.26	0.81	0.83
SD	0.66	3.04	3.57	4.24	0.38	1.02	0.37	0.32
Minimum	7.56	2.71	3.75	1.5	0.71	1.11	0.12	0.35
Maximum	9.42	9.44	12.42	11.74	1.69	4.0	1.19	1.24
Small-scale terapon								
Mean	7.59	7.18	9.15	7.29	1.73	0.98	1.47	1.31
SD	1.16	2.89	1.98	3.07	1.05	0.49	1.25	0.85
Minimum	5.87	1.6	6.3	1.8	0.54	0.34	0.12	0.01
Maximum	8.96	9.17	11.41	10.76	3.12	1.64	3.35	2.41
Permissible limitμg/g (WW)	0.52 ^1^		0.53 ^2^		0.052 ^1^		0.51 ^3^	

^1^ [34], ^2.^[35], ^3^ [36].

**Table 4:** EDI (estimated daily intake) and tolerable daily intake (TDI) of heavy metals.

Fish Name	Pb	Ni	Cd	Mn
Small-scale terapon	0.0006	0.002	0.00027	0.00036
Torpedo scade	0.0007	0.001	0.00051	0.00040
Sicklefish	0.0013	0.001	0.00056	0.00020
Saddle grunt	0.0011	0.001	0.00028	0.00091
Gold silk seabream	0.0020	0.001	0.00043	0.00074
Indian mackerel	0.0020	0.001	0.00024	0.00118
Spotted sickle fish	0.0021	0.002	0.00061	0.00023
Tolerable daily intake (TDI) (mg/kg/day) in fish	0.002	*0.012*	*0.00080*	*0.14000*
Mean EDI (mg/kg/day)	*0.0014*	*0.001*	*0.0004*	*0.0006*
Maximum permissible limit (mg/kg wet weight) in Fish	*2 (a)*	*0.5–1 (a)*	*2 (b)*	*1 (a)*

(a): [35], (b): [36].

**Table 5:** Target hazard quotient (THQ), hazard index (HI), and target cancer risk (TR) from heavy metals in each fish species.

	THQ				HI	Target Cancer Risk (TR)
Fish Name	Pb	Ni	Cd	Mn	(Hazard Index)	Ni
Small-scale terapon	0.17	1.07 *	2.86 *	0.2736	4.374	3.7^−5^
Torpedo scade	0.21	0.44	5.46 *	0.3112	6.437	1.5^−5^
Sicklefish	0.40	0.61	6.02 *	0.1525	7.190	2.1^−5^
Saddle grunt	0.33	0.51	2.98 *	0.7040	4.519	1.8^−5^
Gold silk seabream	0.60	0.65	4.59 *	0.5703	6.415	2.3^−5^
Indian mackerel	0.61	0.64	2.63 *	0.9066	4.784	2.2^−5^
Spotted sickle fish	0.63	0.88	6.61 *	0.1734	8.294	3.1^−5^
Non-carcinogenic Reference Dose (RfD) for fish (mg/kg-day)	0.04 (ASTDR)	0.02 (IRIS)	0.001 (IRIS)	0.014 (IRIS)	THQ > 1, may cause potential health risk HI > 1, adverse health effects are expected TR from 10^−4^ to 10^−6^ (acceptable range) BAF > 1 > 100, potential accumulation BAF > 100 > 1000, significant accumulation BAF > 1000, hazardous accumulation	

* Values > 0.01. ASTDR: Agency for Toxic Substances and Disease Registry. IRIS: Integrated Risk Information System.

**Table 6:** Comparison of this study with other regional studies. Values are in μg/g (ww: wet weight and Dw: dry weight).

Locations	Pb	Ni	Cd	Mn	Reference
Red Sea (Mining Site) (ww)	0.2	5.33	-	9.34	[45]
Mining Area in Brazil (Muscles) (ww)	2.1	-	-	13.0	[46]
Gulf of Aqaba, Red Sea (ww) (Muscles)	4.52	2.20	0.66	0.96	[47]
Jedda coast (Dw)	6.1	_	1.06	-	[48]
South West coast of India (ww)	1.5	0.41	0.11	0.4	[49]
Bangladesh (Bangshi River) (ww)	4.64	2.59	0.3	23.7	[50]
Iran (Mian kaley Lake) (ww)	0.67	0.21	0.26	-	[51]
Saint Martin Island (ww)	0.11–8.92	-	1.52–14.09	0.59–0.74	[52]
Mediterranean Sea (Dw)	7.33–9.11	4.25–6.07	1.07–1.43	-	[53]
Bay of Bengal (Dw)	1.67–2.58	6.43–7.57	0.01–0.16	5.00–11.14	[54]
Palk Bay India (Dw)	0.1–0.12	-	0.02–0.28	-	[55]
Turkish Sea (ww)	0.15–1.15	0.01–3.43	0.01–0.43	0.07–3.62	[56]
Gadani Shipbreaking Area					
Average Concentration (Gills and Muscles)	4.51	5.27	1.33	2.29	This study
